# Supplementary material for: Fetal-derived macrophages dominate in adult mammary glands
Source: Nat Commun. 2019 Jan 17;10:281. doi: 10.1038/s41467-018-08065-1 (PMC6336770; doi:10.1038/s41467-018-08065-1)
Supplement: Supplementary file 1 — Supplementary Information [file 41467_2018_8065_MOESM1_ESM.pdf]

Supplementary Data for

Fetal-derived macrophages dominate in adult mammary glands

Norma Jäppinen, Inês Félix, Emmi Lokka, Sofia Tyystjärvi, Anne Pynttäri, Tiina Lahtela, Heidi Gerke, Kati Elima, Pia Rantakari and Marko Salmi

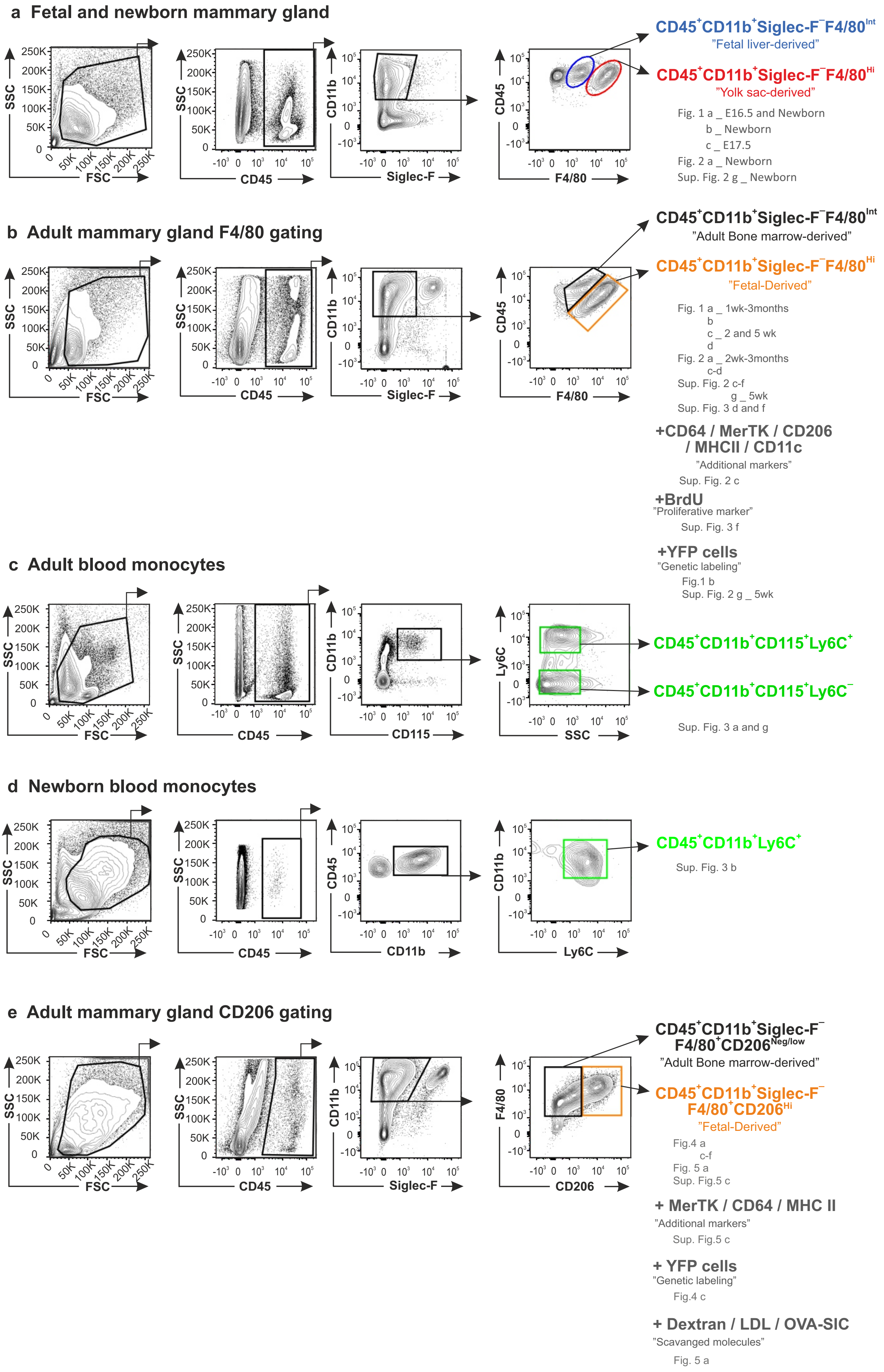

Supplementary Figure 1

Supplementary Figure 1. Gating strategies for flow cytometry

a, b Representative FACS plots showing the gating for F4/80^{Int} and F4/80^{Hi} mammary gland (MG) macrophages in fetal and newborn (**a**) and in 1 wk-3 months old (**b**) mice. **c,d** Representative plots showing the gating for Ly6C^{+} and Ly6C^{-} blood monocytes in adults (**c**) and newborns (**d**). **e** Representative plots showing the gating for CD206$^{Neg/low}$ and CD206^{Hi} macrophages in MG of adult mice. In embryos and newborns, the CD45^{+}CD11b^{+}Siglec-F^{-} F4/80^{Hi} macrophages (phenotypically YS -derived) are gated in red, and the CD45^{+}CD11b^{+}Siglec-F^{-} F4/80^{Int} macrophages (phenotypically fetal liver -derived) in blue. In 1 wk-3 months old mice the CD45^{+}CD11b^{+}Siglec-F^{-} F4/80^{Hi} and CD45^{+}CD11b^{+}Siglec-F^{-} CD206^{Hi} macrophages (both phenotypically fetal-derived) are gated in orange, and the CD45^{+}CD11b^{+}Siglec-F^{-} F4/80^{Int} and CD45^{+}CD11b^{+}Siglec-F^{-} CD206$^{Neg/low}$ macrophages (both phenotypically adult-derived) in black. Blood monocyte populations are gated in green. The individual figure panels using the given gating strategy and coloring code are specified below each population designation.

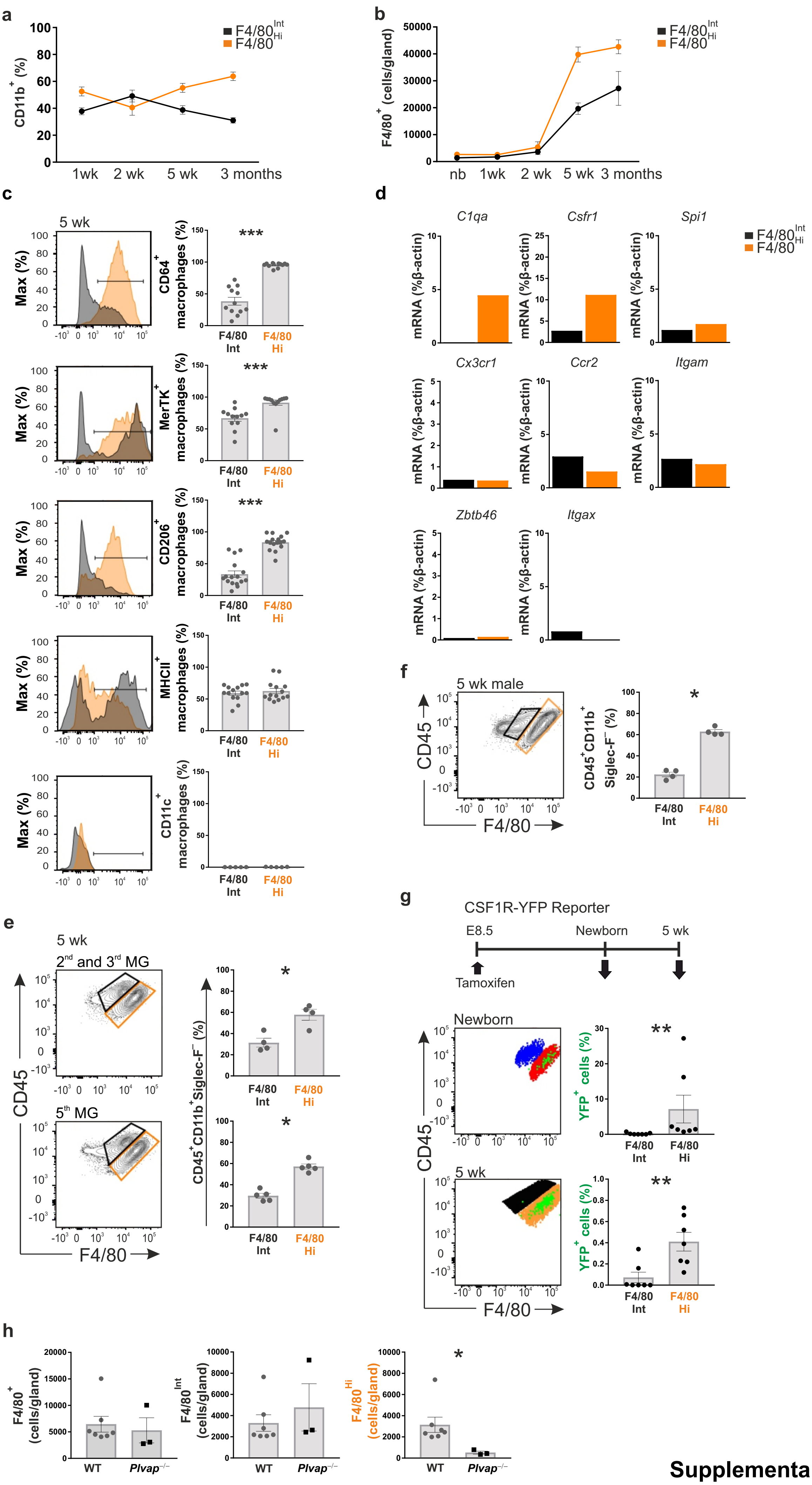

Supplementary Figure 2

Supplementary Figure 2. Leukocyte populations in the experimental mouse models.

a Kinetics of F4/80^{Int} and F4/80^{Hi} macrophage populations in wild type (WT) mammary gland (MG) after birth. **b** Numbers of F4/80^{Int} and F4/80^{Hi} macrophages in the MG of WT mice after birth. **c** Analysis of CD64, MerTK, CD206, MHCII, and CD11c expression on F4/80^{Int} and F4/80^{Hi} MG macrophages in 5 wk old WT mice. **d** qPCR analysis of the indicated transcripts in F4/80^{Int} and F4/80^{Hi} macrophage populations sorted from 5 wk old WT mice. The sorted populations were pooled from 5 donors, and the gene expression values are shown in relation to a control gene β-actin. **e** Flow cytometric analysis of F4/80^{Int} and F4/80^{Hi} macrophage populations in the 2nd and 3rd (pooled) and 5th MG of 5 wk old female WT mice. **f** Flow cytometric analyses of the MG resident macrophages in 5 wk old WT male mice. **g** F4/80^{Int} and F4/80^{Hi} MG macrophages from CSF1R-YFP reporter mice (tamoxifen induction at E8.5) and quantification of YFP^+ cells. The representative FACS plots show backgating of the YFP^+ cells (green) onto F4/80^{Int} macrophages (blue in newborn and black in 5 wk old mice) and F4/80^{Hi} macrophages (red in newborn and orange in 5 wk old mice). The quantifications show the frequency of YFP^+ cells in each macrophage population. **h** Analysis of total F4/80^+ cell number and F4/80^{Int} and F4/80^{Hi} macrophage numbers in MG of 5 wk old WT and *Plvap*$^{-/-}$ mice.

In all panels (**a-h**) MG macrophages were pre-gated as live $CD45^+CD11b^+$Siglec-F^- cells. In the quantifications, each dot represents one mouse and mean ±SEM are shown. Data are from 3 (**a** 1 wk and 5 wk**, b** 1wk**, c** MerTK, MHCII and CD11c**, g** 5 wk, **h**), 2 (**a** 2 wk and 3 months**, b** newborn, 2 wk and 5wk, **d** for sorting, **e**, **g** newborn), 4 (**c** CD64, CD206) and 1 (**f**) independent experiments. * $p<0.05$, ** $p<0.01$, *** $p<0.001$ (Kruskal-Wallis test). Source data are provided as a Source Data file.

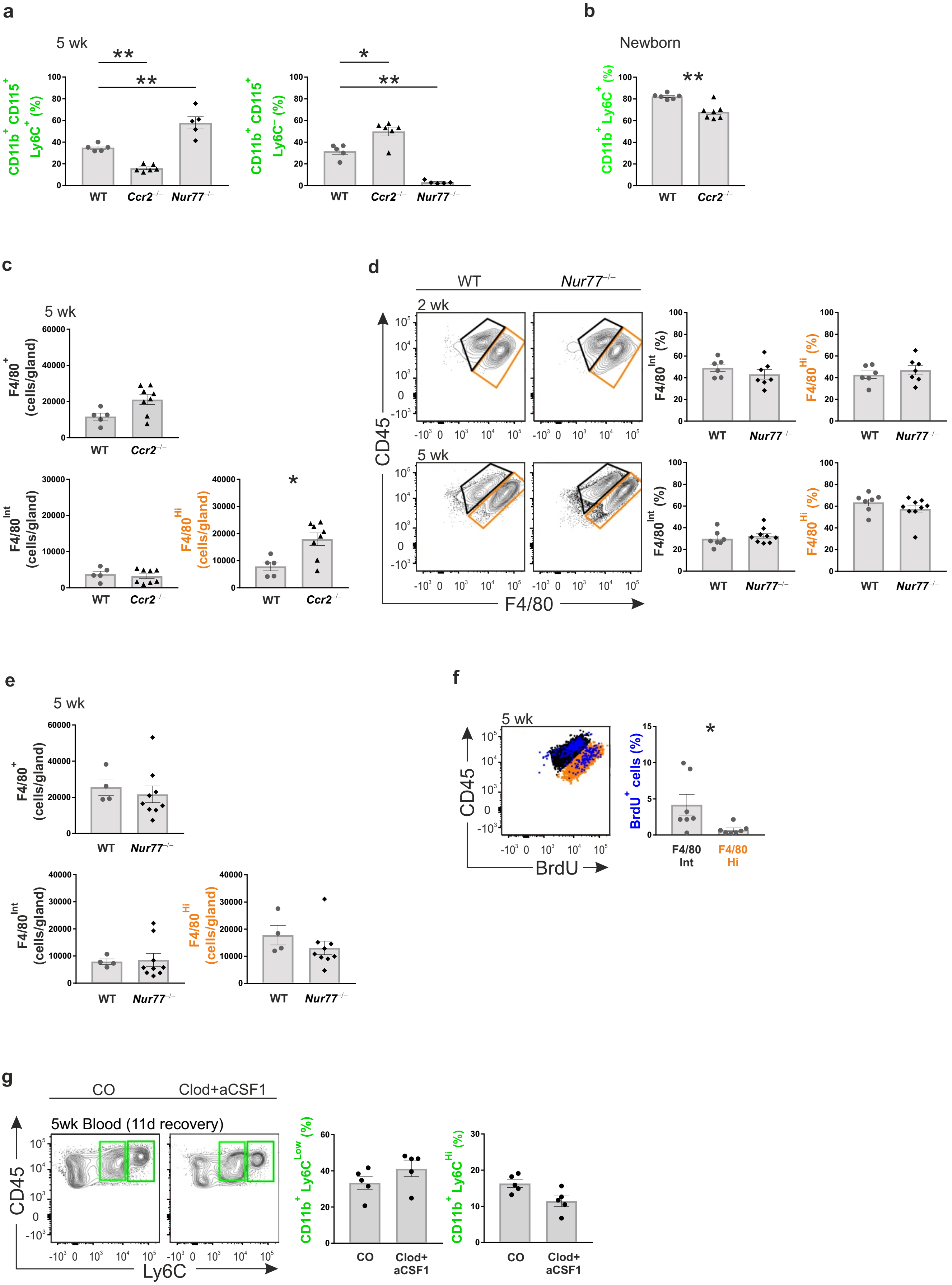

Supplementary Figure 3

Supplementary Figure 3. Mammary gland macrophage populations in gene-deficient mice and after macrophage depletion.

a Ly6C^{+} and Ly6C^{-} blood monocytes in 5 wk old wild type (WT), *Ccr2*$^{-/-}$ and *Nur77*$^{-/-}$ mice. **b** Ly6C^{+} blood monocytes in newborn WT and *Ccr2*$^{-/-}$ mice. **c** Quantification of the total F4/80^{+} cell number and F4/80^{Int} and F4/80^{Hi} macrophage numbers in mammary glands (MG) of 5 wk old WT and *Ccr2*$^{-/-}$ mice. **d** F4/80 expression on MG macrophages in WT and *Nur77*$^{-/-}$ mice and quantification of the frequency of F4/80^{Int} and F4/80^{Hi} populations. **e** Quantification of the total F4/80^{+} cell number and F4/80^{Int} and F4/80^{Hi} macrophage numbers in MG of 5 wk old WT and *Nur77*$^{-/-}$ mice. **f** F4/80^{Int} and F4/80^{Hi} MG macrophages from 5 wk old WT mice pulse-chased with bromodeoxyuridine (BrdU) or not (CO), and the quantification of BrdU+ cells in the MG. The representative FACS plots show backgating of the BrdU^{+} cells (blue) onto F4/80^{Int} macrophages (black cells) and F4/80^{Hi} macrophages (orange cells). The quantifications show the frequency of BrdU^{+} cells in each macrophage population. **g** Ly6C^{Low} and Ly6C^{Hi} blood monocytes in the control (CO) and clodronate-anti-CSF1 treated (Clod+aCSF1) treated mice at 5 wk (after an 11 day recovery period).

In **c-f** MG macrophages were pre-gated as live CD45^{+}CD11b^{+}Siglec-F^{-} cells. In the quantifications, each dot represents one mouse and mean ±SEM are shown. Data are from 2 (**a,d,e,f,g),** 1 **(b)**, and 3 (**c**) independent experiments. * $p<0.05$, ** $p<0.01$, ***$p<0.001$ (Kruskal-Wallis test). Source data are provided as a Source Data file.

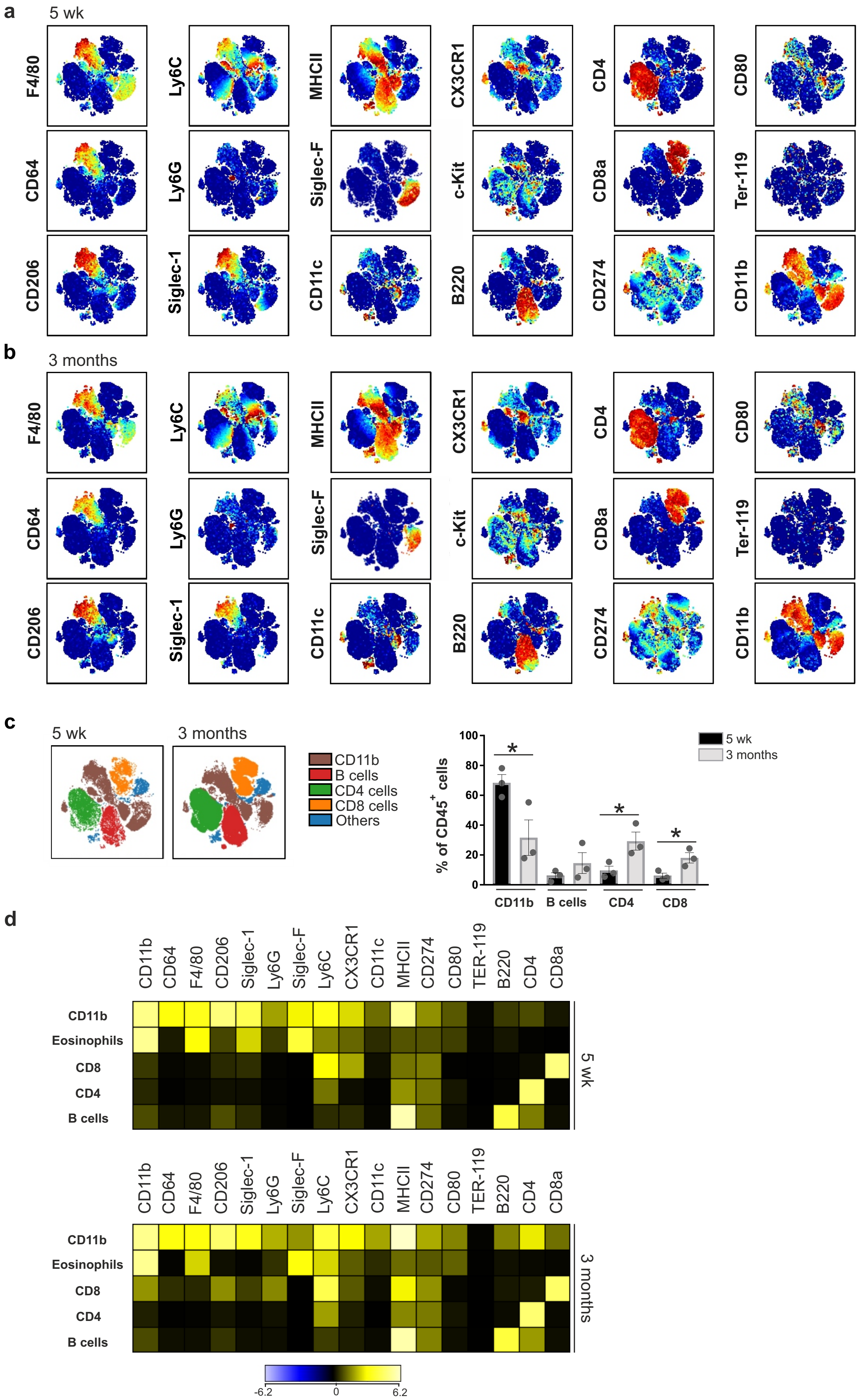

Supplementary Figure 4

Supplementary Figure 4. Mass cytometric analyses of tissue-resident mammary gland leukocytes .

a-c Unsupervised analyses of randomly sampled $CD45^+$ leukocytes from mammary glands (MG) of 5 wk and 3 months old wild type (WT) mice. The expression intensity of the indicated markers in 5 wk old mice (**a**) and 3 months old (**b**) mice is displayed on the t-SNE maps. **c** Frequencies of resident $CD45^+$ leukocytes in the MG in steady state. Based on the unsupervised expression analyses of all leukocyte subtype selective markers (**a,b**), coloring was manually added to the t-SNE maps to visualize different cell types. The frequencies of each cell type from all $CD45^+$ leukocytes is shown as mean ±SEM (each dot represents one mouse) are shown * $p<0.05$, ** $p<0.01$, ***$p<0.001$ (Kruskal-Wallis test). **d** Mass cytometric analysis of mean marker expression for the indicated leukocyte populations in the MG of 5 wk and 3 months old WT mice.

All data are from 3 independent experiments. Source data are provided as a Source Data file.

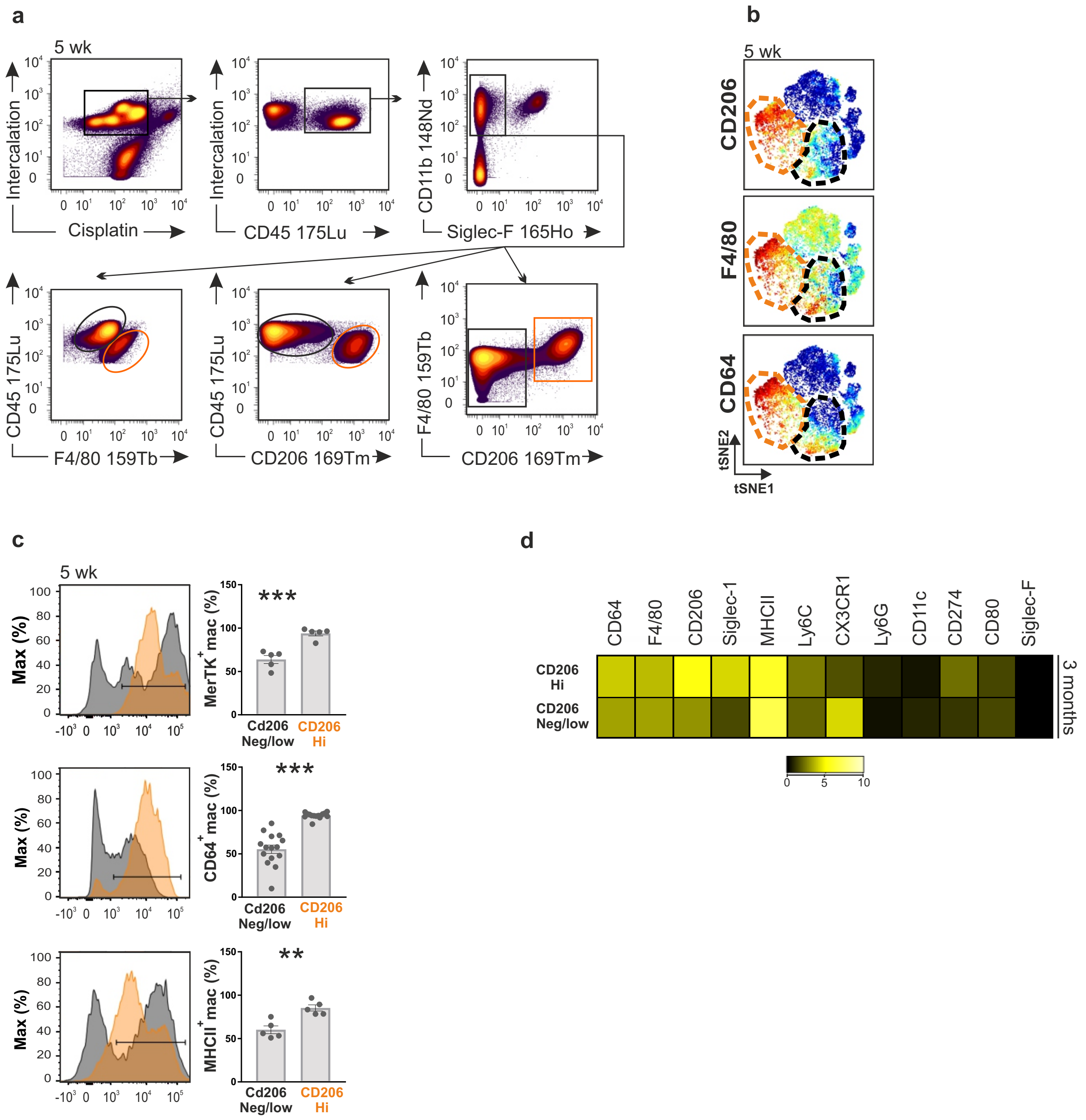

Supplementary Figure 5

Supplementary Figure 5. Analyses of mammary gland macrophages

a Manual bi-axial gating of the mass cytometric data for live (Cisplatin^{-}) singlet (intercalation^{+}) CD45^{+}SiglecF^{-}CD11b^{+} cells from mammary glands (MG) of wild type (WT) mice for analyses of F4/80 and CD206 expression. **b** Unsupervised analyses of the mass cytometric data (t-SNE maps) displaying randomly sampled CD45^{+}CD11b^{+} myeloid cells from the MG for expression of CD206 and F4/80 in 5 wk old WT mice. The manually drawn circles show the approximation of F4/80^{Int} (black dotted line) and F4/80^{Hi} (orange dotted line) macrophage phenotypes observed in fluorimetric flow cytometry on the mass cytometric macrophage populations. These data are from a different mouse than those displayed in Fig. 3a. **c** Fluorimetric analysis of MerTK, CD64 and MHCII expression in CD206$^{Neg/low}$ and CD206^{Hi} macrophage populations in MG of 5 wk old WT mice. MG macrophages were pre-gated as live CD45^{+}CD11b^{+}Siglec-F^{-} cells. In the quantifications, each dot represents one mouse and mean ±SEM are shown. * $p<0.05$, ** $p<0.01$, *** $p<0.001$ (Kruskal-Wallis test). **d** Mass cytometric analysis of mean marker expression for CD206^{Hi} and CD206$^{Neg/low}$ macrophage subsets in the MG of 3 months old WT mice.

Data are from 1(**b**), 2 (**c**, MerTK, MHCII) and 3 (**c** CD64 and **d**) independent experiments. Source data are provided as a Source Data file.

a

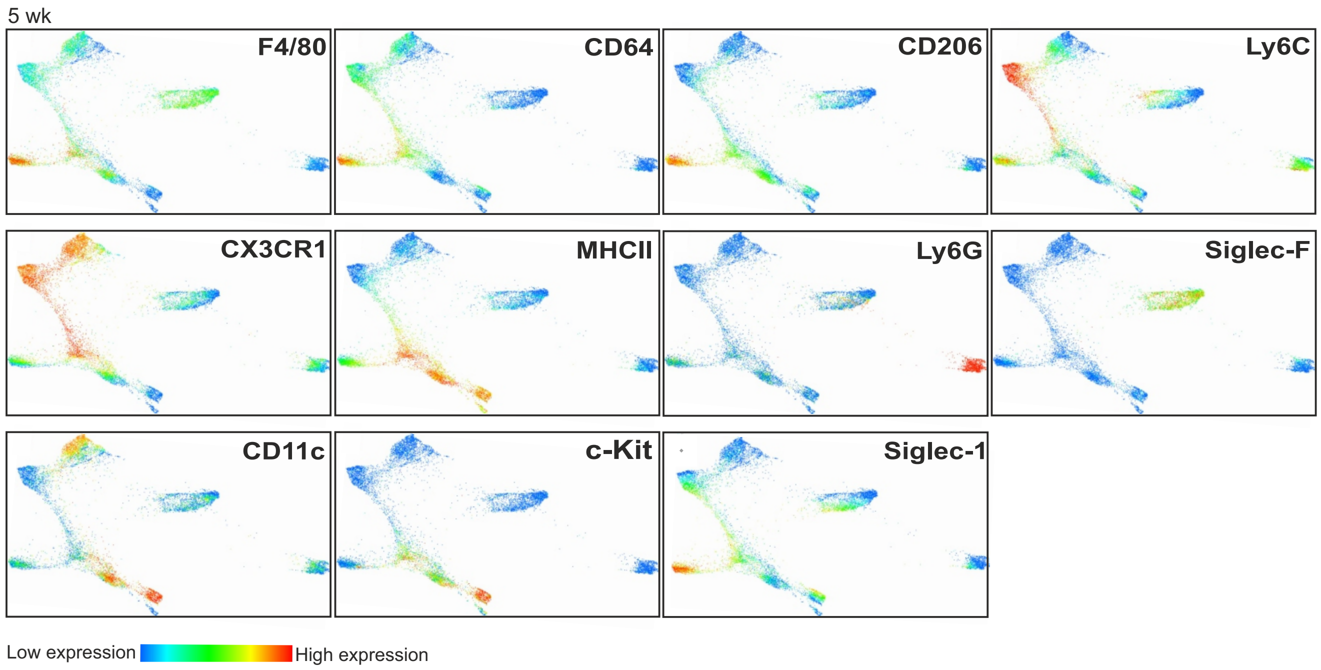

b

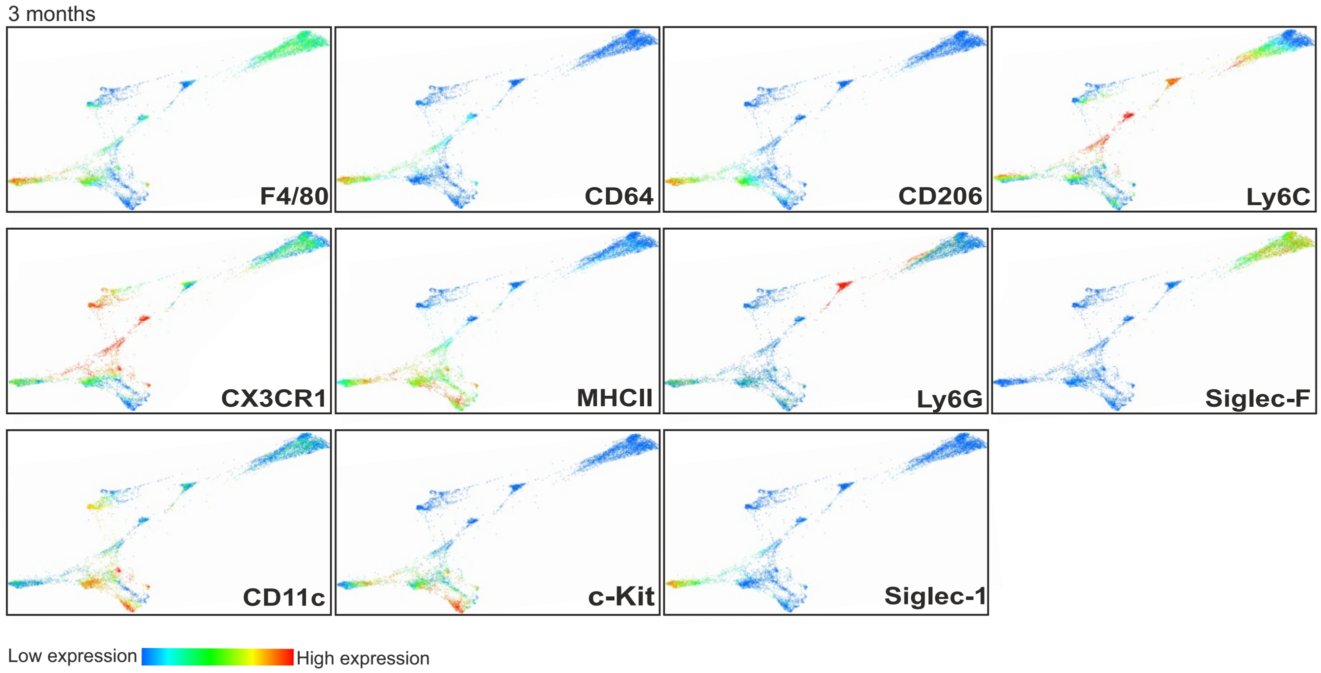

c

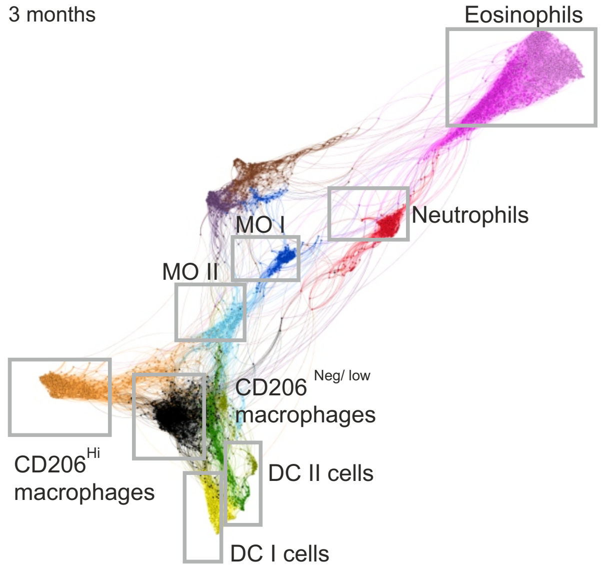

Supplementary Figure 6

Supplementary Figure 6. X-shift clustering analyses of macrophage development in mammary glands

a Unsupervised hierarchial X-shift clustering (nearest neighbor) of $CD45^+CD11b^+$ myeloid cells in mammary glands (MG) of 5 wk old wild type (WT) mice with superimposed with expression analyses of the indicated leukocyte differentiation markers. Color code represents expression levels of the indicated markers. The expression patterns of the given markers were used for manual designation of the cell clusters in Fig. 3e. **b** Unsupervised hierarchial X-shift clustering of $CD45^+CD11b^+$ myeloid cells in MG of 3 months old WT mice with superimposed with expression analyses of the indicated leukocyte differentiation markers. **c** Single-cell force-directed layout of MG $CD45^+CD11b^+$myeloid cells in 3 months old WT mice. Based on the expression analyses of all leukocyte subtype-selective markers (**b**), gray boxes and coloring were manually added to visualize the identity of different clusters.

All data are representative of 3 independent experiments.

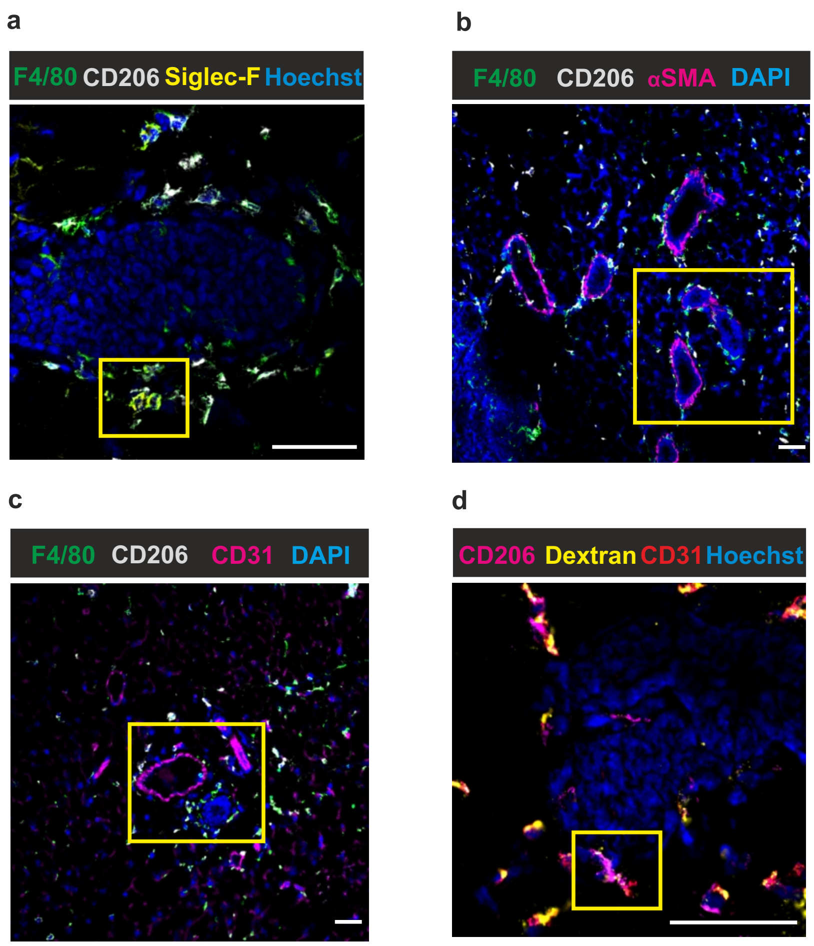

Supplementary Figure 7. Distinct fetal-derived CD206^{Hi} and adult-derived CD206$^{Neg/low}$ macrophages subsets in adult mammary glands.

a Immunohistochemical analysis of CD206, F4/80, and Siglec-F expression in the mammary glands (MG) of 5 wk old wild type (WT) mice. Fig. 5c is a higher magnification from the boxed area. Blue is Hoechst. Bar, 25 μm. **b** Immunohistochemical analysis of CD206, F4/80 and αSMA expression in the MG. Blue is DAPI. Fig. 5d is a higher magnification from the boxed area. Bar, 50 μm. **c** Immunohistochemical analysis of CD206, F4/80 and CD31 expression in the MG. Blue is DAPI. Fig. 5e is a higher magnification from the boxed area. Bar, 50 μm. **d** Microscopic analyses of CD206 and CD31 in MG of mice injected intravenously with fluorescent dextran (yellow). Fig. 5f is a higher magnification from the boxed area. Blue is Hoechst. Bar, 25 μm. Data are representative of 3 independent experiments.

Supplementary Table 1. Antibodies used in this study

Antibodies			Dilutions/Concentrations		
			Flow	CyTOF	Immunohistochemistry or In Vivo
Anti mouse aSMA-FITC	Sigma	cat# F3777-2ML			1:500
Anti mouse B220-159Tb	Fluidigm	cat# 3159015C		1:200	
Anti mouse CD4-172Yb	Fluidigm	cat# 3172003C		1:200	
Anti mouse CD8a-168Er	Fluidigm	cat# 3168003C		1:200	
Anti mouse CD11b-148Nd	Fluidigm	cat# 3148003C		1:200	
Anti mouse CD11b-PE	BD Biosciences	cat# 553311	1:400		
Anti mouse CD11b-APC-Cy7	BD Biosciences	cat# 557647	1:400		
Anti mouse CD11b-BB515	BD Biosciences	cat# 564454	1:400		
Anti mouse CD11b-BV786	BD Biosciences	cat# 740861	1:400		
Anti mouse CD11c-142Nd	Fluidigm	cat# 3142003C		1:200	
Anti mouse CD11c –BV421	BD Biosciences	cat# 562782	1:200		
Anti mouse CD16/CD32 (2.4G2)	Bio X Cell	cat# CUSTOM24G2	1.5:100		
Anti mouse CD31 (MEC13.3)	BD Biosciences	cat# 553370			10µg/ml
Anti mouse CD31-APC	BioLegend	cat# 102514			10µg/ml
Anti mouse CD45-175Lu	Fluidigm	cat# 3175010C		1:200	
Anti mouse CD45-PerCP-Cy5.5	BD Biosciences	cat# 550994	1:200		
Anti mouse CD64-151Eu	Fluidigm	cat# 3151012C		1:200	
Anti mouse CD64-PE	BioLegend	cat# 139304	1:200		
Anti mouse CD80-171Yb	Fluidigm	cat# 3171008C		1:200	
Anti mouse CD115-PE-Cy7	eBioscience	cat# 25-1152-805	1:400		
Anti mouse CD117-173Yb	Fluidigm	cat# 3173004C		1:200	
Anti mouse CD144	Pharmingen	cat# 550548			10µg/ml
Anti mouse CD206-169Tm	Fluidigm	cat# 3169021C		1:200	
Anti mouse CD206-A488	Bio-Rad	cat# MCA2235A488T			10µg/ml
Anti mouse CD206-A647	BD Biosciences	cat# 565250			8µg/ml
Anti mouse CD206-BV650	BioLegend	cat# 141723	1:200		
Anti mouse CD274-153Eu	Fluidigm	cat# 3153016C		1:200	
Anti mouse CSF1 (5A1)	Bio X Cell	cat# BE0204			0.5 or 0.25 mg/injection
Anti mouse CSF1R (AFS98)	Bio X Cell	cat# BE0213			3mg/ injection
Anti mouse CX3CR1-164Dy	Fluidigm	cat# 3164023C		1:200	
Anti mouse F4/80-146Nd	Fluidigm	cat# 3146008C		1:200	
Anti mouse F4/80-A488	eBioscience	cat# 53-4801-82	1:200		
Anti mouse F4/80 (CI:A3-1)	Bio-Rad	cat# MCA497R			10µg/ml
Anti mouse F4/80-A647	Bio-Rad	cat# MCA497A647	1:100		
Anti mouse IgG1 (HRPN)	Bio X Cell	cat# BE0088			0.5 or 0.25mg/injection
Anti mouse IgG2a (2A3)	Bio X Cell	cat# BE0089			3mg/injection
Anti mouse Ly6C-162Dy	Fluidigm	cat# 3162014C		1:200	
Anti mouse Ly6C-BV421	BD Biosciences	cat# 562727	1:200		
Anti mouse Ly6G-141Pr	Fluidigm	cat# 3141008C		1:200	
Anti mouse Ly6G-BV510	BioLegend	cat# 127633	1:200		
Anti mouse MerTK-PE	BioLegend	cat# 151505	1:200	1:100	
Anti mouse MHCII-174Yb	Fluidigm	cat# 3174003C		1:200	
Anti mouse MHCII-PE-Cy7	eBioscience	cat# 25-5321-82	1:400		
Anti PE (PE004) 158HO	Fluidigm	cat# 3165015C		1:200	

Anti mouse Siglec-1-170Er	Fluidigm	cat# 3170018C		1:200		
Anti mouse Siglec-F-A647	BD Biosciences	cat# 562680			10µg/ml	
Anti mouse Siglec-F-PE-CF594	BD Biosciences	cat# 562757	1:200	1:100		
Anti mouse TER-119-154Sm	Fluidigm	cat# 3154005C		1:200		
Anti rat IgG (polyclonal)	ThermoFisher Scientific	cat# A11081			10µg/ml	
