# Supplementary material for: Fetal-derived macrophages dominate in adult mammary glands
Source: Nat Commun. 2019 Jan 17;10:281. doi: 10.1038/s41467-018-08065-1 (PMC6336770; doi:10.1038/s41467-018-08065-1)
Supplement: Supplementary file 3 — Reporting Summary [file 41467_2018_8065_MOESM3_ESM.pdf]

nature research

Corresponding author(s): Marko Salmi NCOMMS-18-28152B

Reporting Summary

Nature Research wishes to improve the reproducibility of the work that we publish. This form provides structure for consistency and transparency in reporting. For further information on Nature Research policies, see Authors & Referees and the Editorial Policy Checklist.

Statistical parameters

When statistical analyses are reported, confirm that the following items are present in the relevant location (e.g. figure legend, table legend, main text, or Methods section).

n/a	Confirmed	
☐	☒	The exact sample size (*n*) for each experimental group/condition, given as a discrete number and unit of measurement
☐	☒	An indication of whether measurements were taken from distinct samples or whether the same sample was measured repeatedly
☐	☒	The statistical test(s) used AND whether they are one- or two-sided<br>*Only common tests should be described solely by name; describe more complex techniques in the Methods section.*
☐	☒	A description of all covariates tested
☐	☒	A description of any assumptions or corrections, such as tests of normality and adjustment for multiple comparisons
☐	☒	A full description of the statistics including central tendency (e.g. means) or other basic estimates (e.g. regression coefficient) AND variation (e.g. standard deviation) or associated estimates of uncertainty (e.g. confidence intervals)
☐	☒	For null hypothesis testing, the test statistic (e.g. *F*, *t*, *r*) with confidence intervals, effect sizes, degrees of freedom and *P* value noted<br>*Give P values as exact values whenever suitable.*
☒	☐	For Bayesian analysis, information on the choice of priors and Markov chain Monte Carlo settings
☒	☐	For hierarchical and complex designs, identification of the appropriate level for tests and full reporting of outcomes
☒	☐	Estimates of effect sizes (e.g. Cohen's *d*, Pearson's *r*), indicating how they were calculated
☐	☒	Clearly defined error bars<br>*State explicitly what error bars represent (e.g. SD, SE, CI)*

Our web collection on statistics for biologists may be useful.

Software and code

Policy information about availability of computer code

Data collection	Spinning disk confocal images were acquired with Slide Book 6 software (Intelligent Imaging Innovations Inc) and confocal microscope images were acquired with Zen 2010 software (Zeiss) . Flow cytometry data was acquired with FACSDiva™ software (BD Bioscience) . Mass flow cytometry data was acquired with CyTOF 6.7 system control software (Fluidigm).
Data analysis	Microscopy data was analyzed by Image J software. Flow cytometry data was analyzed by FlowJo (TreeStar). Mass cytometry data was analyzed with Cytobank (https://www.cytobank.org) or with VorteX clustering environment (https://github.com/nolanlab/vortex/releases/tag/29-Jun-2017; Published in Nat Methods. 2016 June ; 13(6): 493–496. doi:10.1038/nmeth.3863.) Statistical analyses were done using SAS. Custom algorithms or software were not used in this study.

For manuscripts utilizing custom algorithms or software that are central to the research but not yet described in published literature, software must be made available to editors/reviewers upon request. We strongly encourage code deposition in a community repository (e.g. GitHub). See the Nature Research guidelines for submitting code & software for further information.

Data

Policy information about availability of data

All manuscripts must include a data availability statement. This statement should provide the following information, where applicable:

- Accession codes, unique identifiers, or web links for publicly available datasets
- A list of figures that have associated raw data
- A description of any restrictions on data availability

All materials used in this study are available commercially or from the authors. The sources for the materials are specified in the Materials and Methods section.

Field-specific reporting

Please select the best fit for your research. If you are not sure, read the appropriate sections before making your selection.

☒ Life sciences ☐ Behavioural & social sciences ☐ Ecological, evolutionary & environmental sciences

For a reference copy of the document with all sections, see nature.com/authors/policies/ReportingSummary-flat.pdf

Life sciences study design

All studies must disclose on these points even when the disclosure is negative.

Sample size	Pilot analyses and previous literature were used to determine sample size.
Data exclusions	All data from experiments in which the positive and/or negative controls worked appropriately were included in the study. If the positive and/or negative controls did not work, the whole experiment was excluded from the analyses.
Replication	All data are at least from 3-8 individual mice. The exact n-numbers are indicated in each Figure as dots. One dot presents a one mouse.
Randomization	Littermate wild-type mice of the same sex were randomly assigned to the experimental groups. When gene-modified mice were used, age-, sex- and strain-matched wild-types were used as controls.
Blinding	The investigators were not blinded to mouse allocation during experiments and outcome assessments.

Reporting for specific materials, systems and methods

Materials & experimental systems

n/a	Involved in the study	
☒	☐	Unique biological materials
☐	☒	Antibodies
☒	☐	Eukaryotic cell lines
☒	☐	Palaeontology
☐	☒	Animals and other organisms
☒	☐	Human research participants

Methods

n/a	Involved in the study	
☒	☐	ChIP-seq
☐	☒	Flow cytometry
☒	☐	MRI-based neuroimaging

Antibodies

Antibodies used	All antibodies (clones, host species, isotypes, fluorochrome-conjugates, and vendors) have been described in Supplemetary Table 1
Validation	The primary antibodies have been validated for the immunohistochemistry on frozen sections by the vendors or by our previous published work or by the specificity controls (isotype controls) of for the flow cytomerty or mass cytometry by the commercial vendors.

Animals and other organisms

Policy information about studies involving animals; ARRIVE guidelines recommended for reporting animal research

Laboratory animals	Plvap−/− mice have been previously described (Rantakari et al., 2015; Rantakari et al., 2016b). Ccr2−/− (stock 004999), Nur77−/−303 (stock 006187), R26R-EYFP (stock 006148), Cx3cR1-CreERT2 (stock 020940) and Csf1r-Mer-iCre-Mer (stock 019098) mice were purchased from Jackson Laboratories. C57BL/6J and C57BL/6N mice were purchased from Janvier labs.
Wild animals	Not used.
Field-collected samples	Not used.

Flow Cytometry

Plots

Confirm that:

- [ ] The axis labels state the marker and fluorochrome used (e.g. CD4-FITC).
- [x] The axis scales are clearly visible. Include numbers along axes only for bottom left plot of group (a 'group' is an analysis of identical markers).
- [x] All plots are contour plots with outliers or pseudocolor plots.
- [x] A numerical value for number of cells or percentage (with statistics) is provided.

Methodology

Sample preparation	The sample preparation has been described in detail in the " Flow cytometry and cell sorting " and "Mass cytometry" sections of the Materials and Methods.
Instrument	The cells were analyzed using a BD Fortessa flow cytometer or FACS aria II cell sorter which were calibrated using compensation beads for each different antibody cocktail or with CyTOF mass cytometer (Helios, Fluidigm).
Software	All flow cytometric data were analyzed using Flow Jo software (TreeStar). Mass cytometry data was either analysed and visualized with Cytobank (https://www.cytobank.org) or analyzed with VorteX clustering environment (https://github.com/nolanlab/vortex/releases/tag/29-Jun-2017)and visualized with Gephi 0.9.1 (https://gephi.org).
Cell population abundance	The abundance of the cell populations is given as percentages.
Gating strategy	The gating strategies and the gate boundaries are shown in the Figures and in the Materials and Methods.

- [x] Tick this box to confirm that a figure exemplifying the gating strategy is provided in the Supplementary Information.
